# Supplementary material for: The risk of pathogenicity and antibiotic resistance in deep-sea cold seep microorganisms
Source: mSystems. 2025 May 21;10(6):e01571-24. doi: 10.1128/msystems.01571-24 (PMC12172429; doi:10.1128/msystems.01571-24)
Supplement: Supplemental Figures — Figures S1 to S15. [file msystems.01571-24-s0001.docx]

*Supplementary Information*

**The risk of pathogenicity and antibiotic resistance in deep-sea**

**cold seep microorganisms**

Tianxueyu Zhang^1, 2^, Yingchun Han^3^, Yongyi Peng^3, 4^, Zhaochao Deng^5, 6^, Wenqing Shi^7, 8^, Xuewei Xu^2^, Yuehong Wu^1, 2 #^, Xiyang Dong^3 #^

^1^ School of Oceanography, Shanghai Jiao Tong University, Shanghai 200030, China

^2^ State Key Laboratory of Submarine Geoscience, Second Institute of Oceanography, Ministry of Natural Resources, Hangzhou 310005, China

^3^ Key Laboratory of Marine Genetic Resources, Third Institute of Oceanography, Ministry of Natural Resources, Xiamen 361005, China

^4^ Department of Microbiology, Biomedicine Discovery Institute, Monash University, Clayton, VIC 3800, Australia

^5^ Institute of Marine Biology and Pharmacology, Ocean College, Zhejiang University, Zhoushan 316021, China

^6^ Ocean Research Center of Zhoushan, Zhejiang University, Zhoushan 316021, China

^7^ State Key Laboratory for Marine Environmental Science, Institute of Marine Microbes and Ecospheres, College of Ocean and Earth Sciences, Xiamen University, Xiamen 361102, China

^8^ RU Marine Symbioses, RD3 Marine Ecology, GEOMAR Helmholtz Centre for Ocean Research Kiel, Kiel 24148, Germany

# Correspondence can be addressed to Xiyang Dong (dongxiyang@tio.org.cn) or Yuehong Wu (yuehongwu@sio.org.cn).

**This additional information contains:**

- - 16 Pages
  - 15 Figures


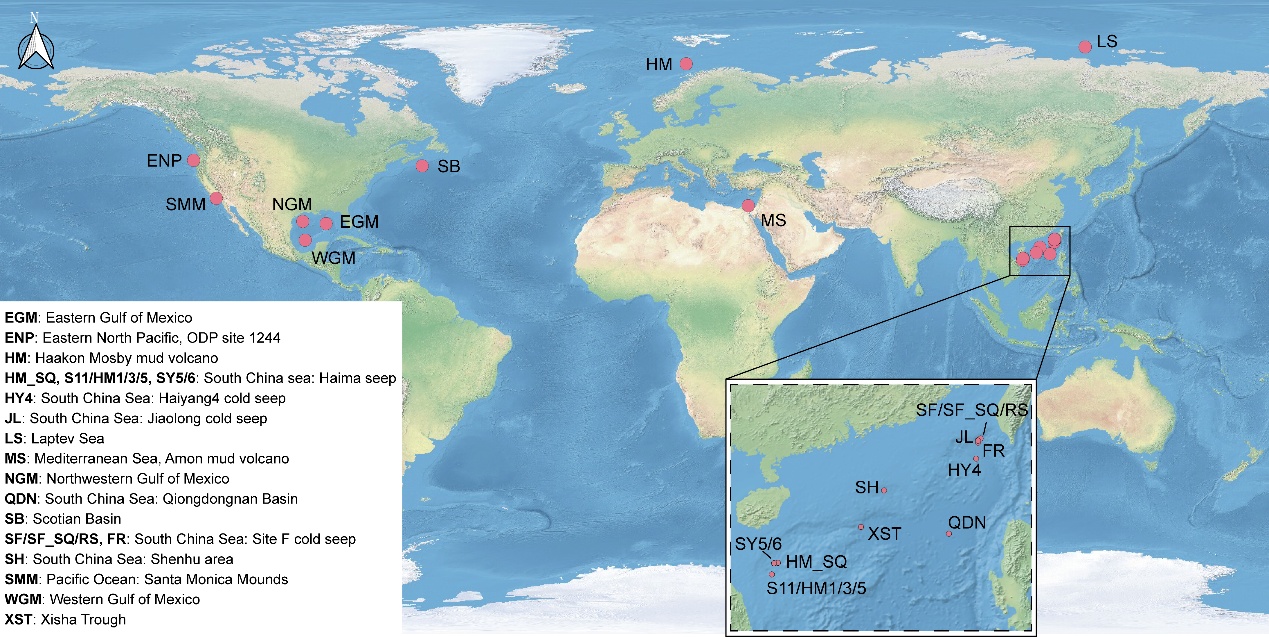


**Fig. S1. Geographic distribution of the 16 global cold seep sites where 165 metagenomic and 33 metatranscriptomic sequencing data were collected.** All datasets used in this study were obtained from our previous publication (1). Details are shown in **Table S1**.


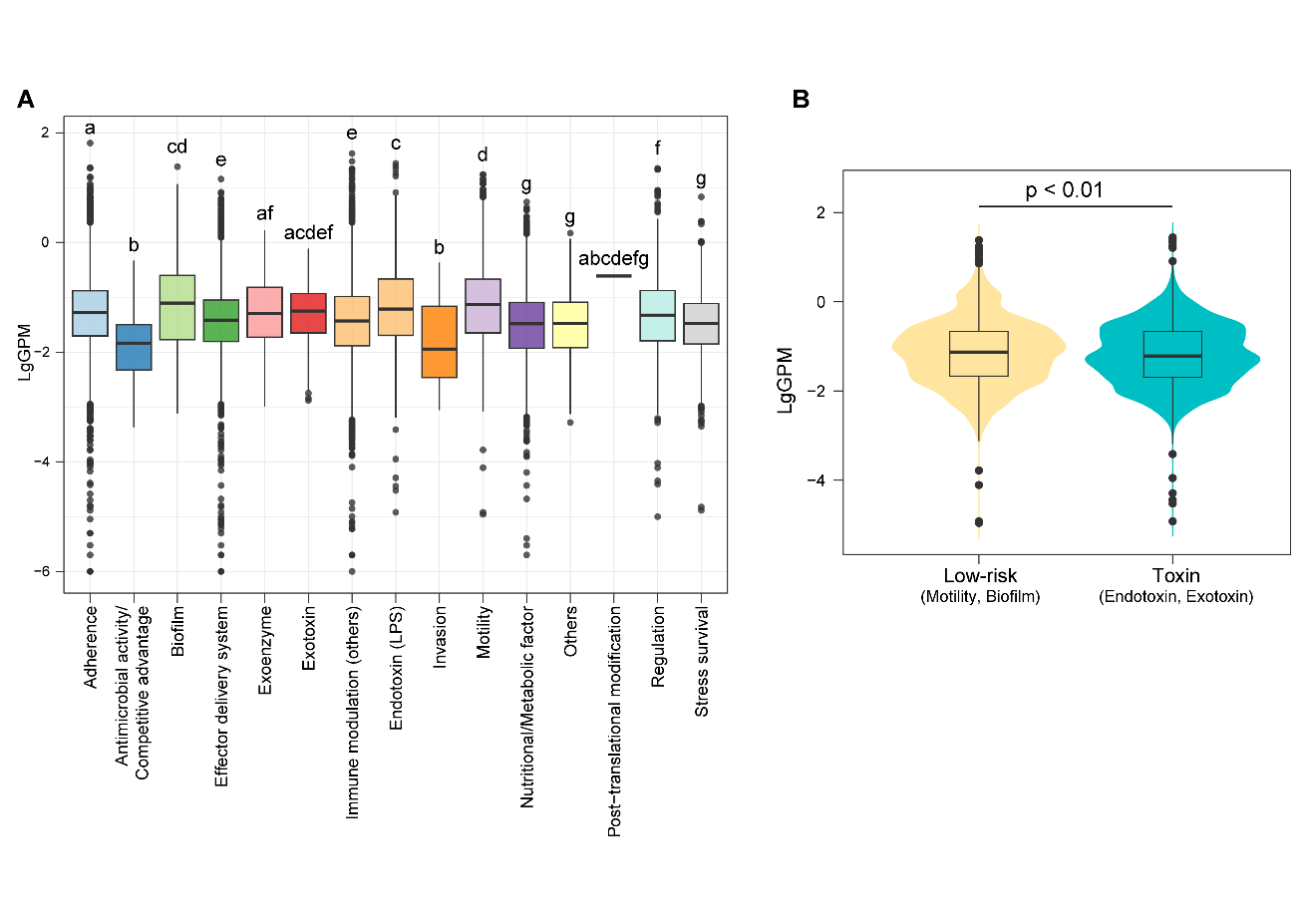


**Fig. S2. Significant differences of the abundance of different VFs.** **(A)** The boxplot comparing the abundance of VFs across different categories. Significance of letters is labelled based on p < 0.05. **(B)** The difference between high-risk (exotoxins and endotoxins) and low-risk (biofilm and motility) VFs.


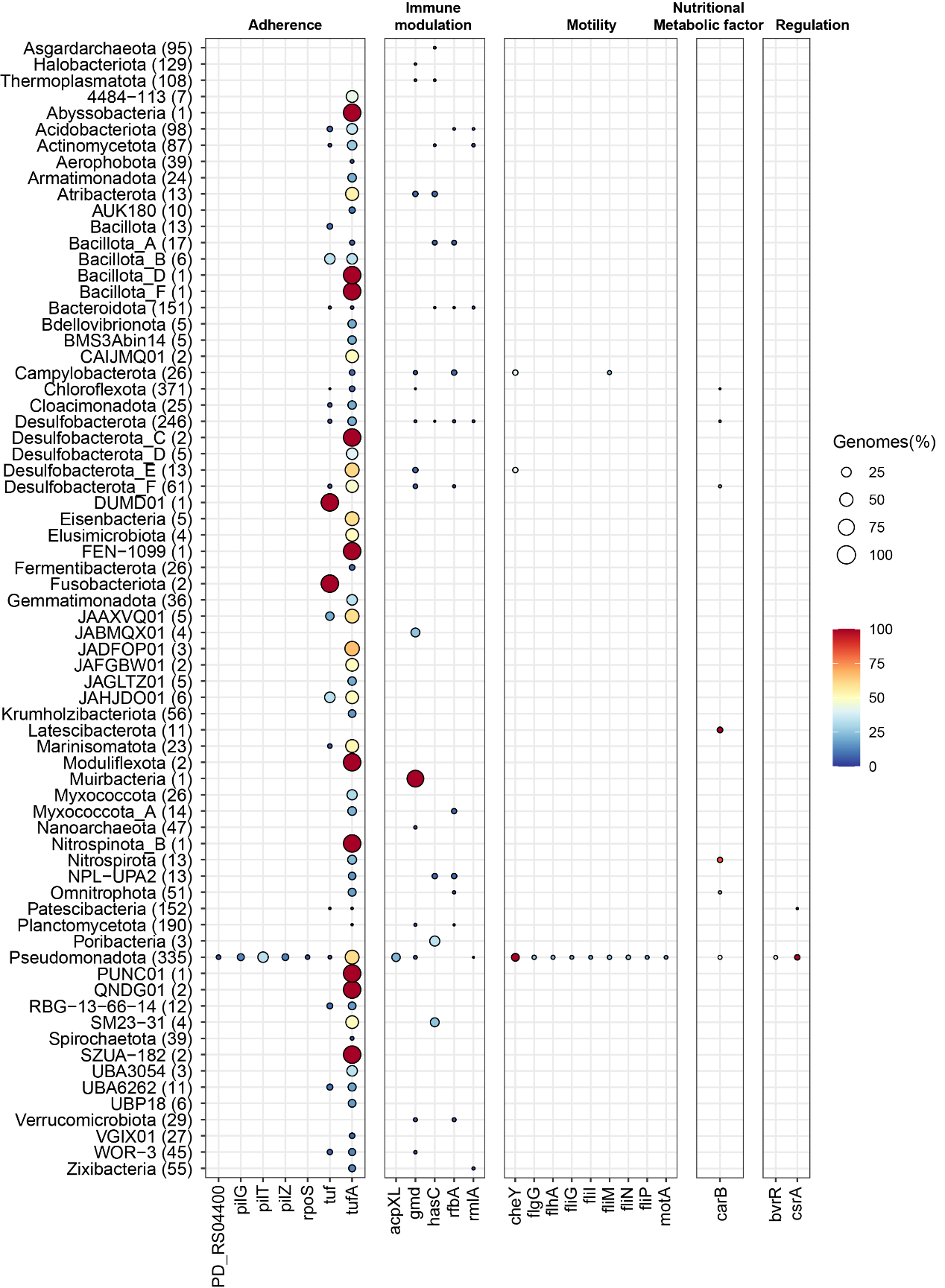


**Fig. S3. Percentage of some abundant VF genes** **encoded within each phylogenetic cluster.** The number of MAGs in each phylogenetic cluster is indicated in brackets. The size of the circles represents the percentage of genomes within each cluster containing the VF gene, and the color gradient indicates the percentage range (from 0 to 100%, as shown in the legend).


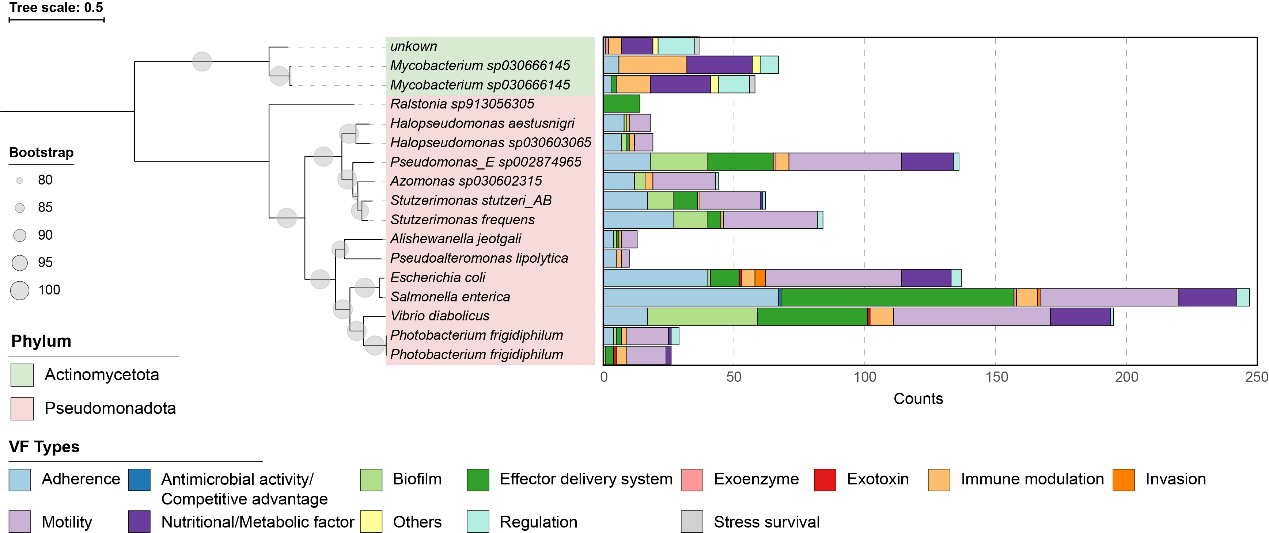


**Fig. S4. Counts of different VF types in VF-riched MAGs.** The phylogenetic tree on the left, constructed based on a set of 120 conserved single-copy marker genes, shows the relationship between different species within the *Actinomycetota* (green) and *Pseudomonadota* (pink) phyla. Bootstrap values are indicated by the size of the gray circles at each node. Bar plot on the right display the counts of VF types within each microbial taxa.


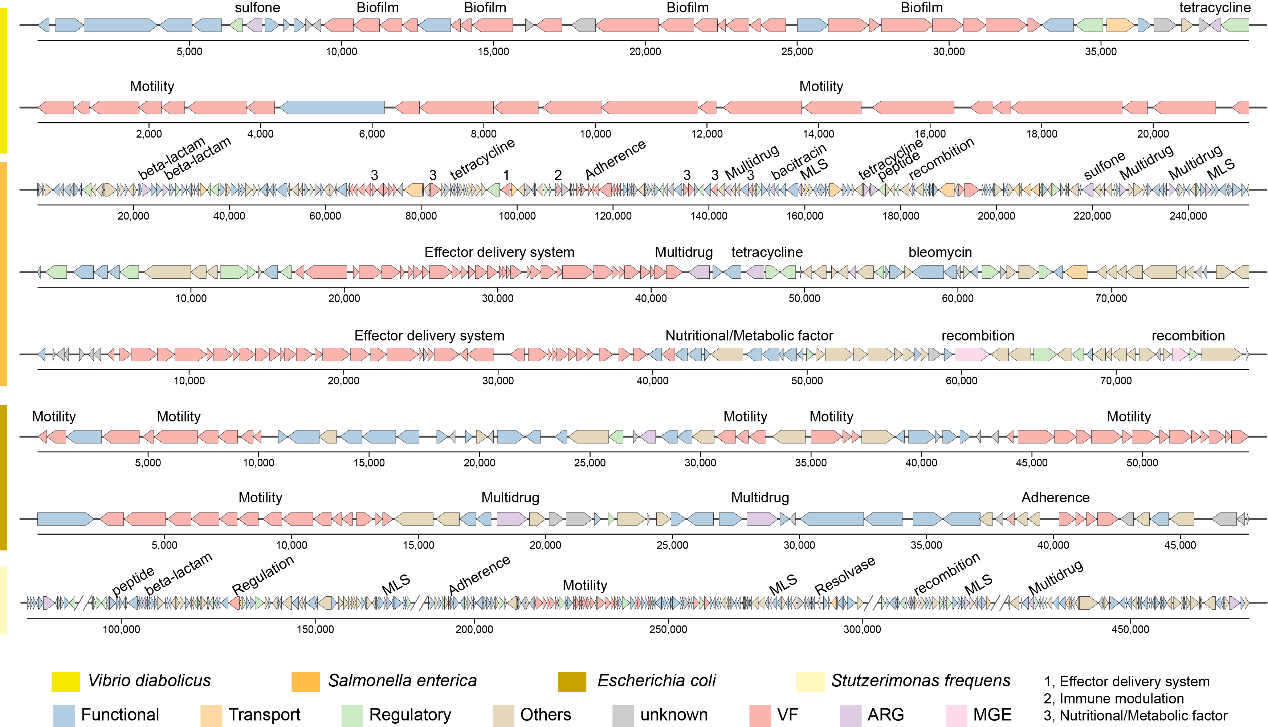


**Fig. S5. Co-localization of VF genes with neighboring genes in VF-rich contigs.** Genes are color-coded by function, including functional genes, transport, regulatory elements, VFs, ARGs, MGEs, and others. The colors on the left indicate the species to which each contig belongs.


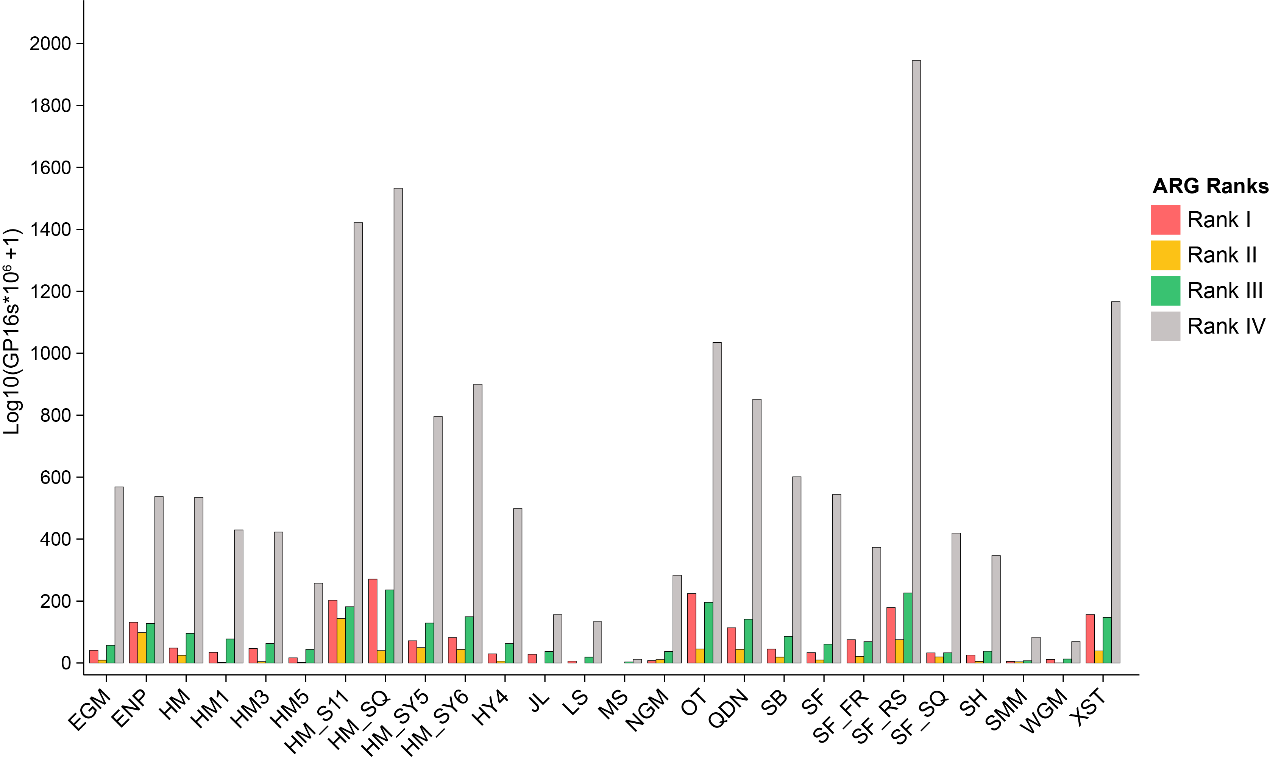


**Fig. S6. Risk assessment of ARGs in cold seep sediments.** Human-associated, mobile ARGs were classified into high-risk categories by ARGranker. The classification differentiates between “current threats” (Rank I), which represent the highest risk of dissemination among pathogens, and “future threats” (Rank II), which indicate a high potential for the emergence of new resistance in pathogens. Abundance was log-transformed (i.e., Log10(GP16S × 10^6^ + 1)) for the plot. The x-axis represents the abbreviations of cold seep sites.


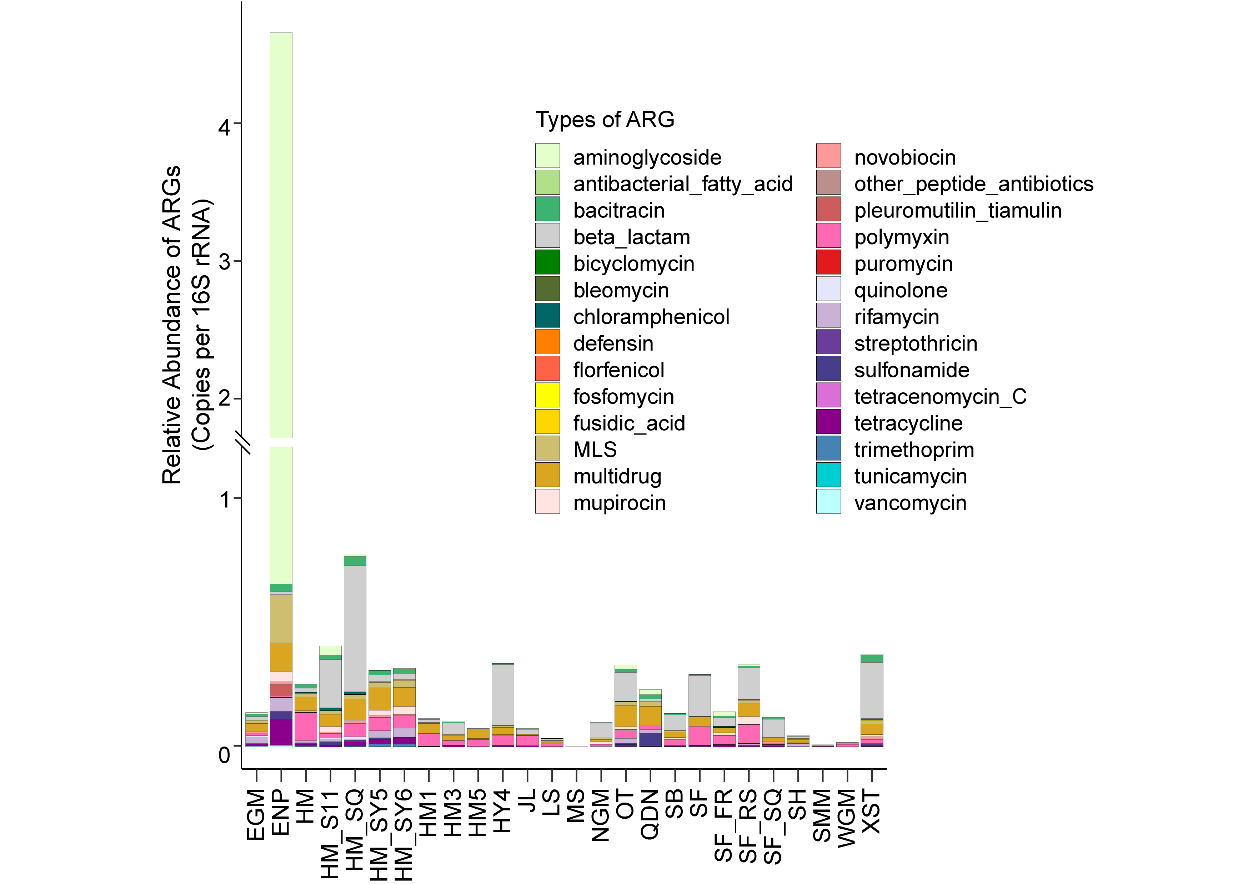


**Fig. S7. Relative abundances of ARGs across different sites.** The x-axis represents the abbreviations of cold seep sites. Relative abundance of ARGs, normalized to the 16S rRNA gene, was reported as ‘gene copies per 16S rRNA gene (GP16S)’. Details are shown in **Table S6**.


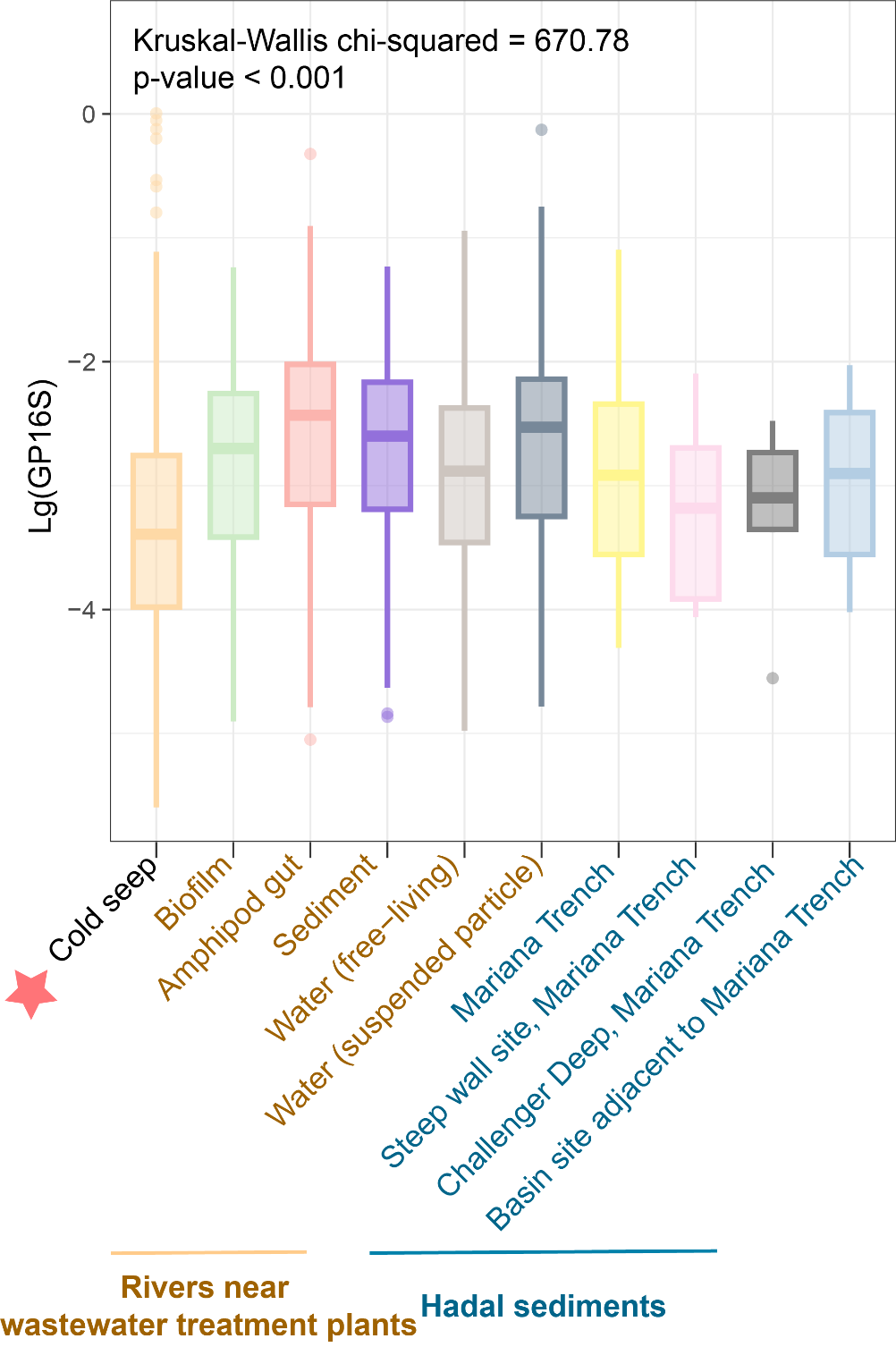


**Fig. S8. Comparison of ARG abundances across various habitats.** Abundances were log-transformed (Log10(GP16S + 1)) for plotting, where GP16S represents gene copies per 16S rRNA gene. The comparison includes samples from rivers near wastewater treatment plants (upstream and downstream), covering habitats such as river water, sediment, biofilm, and amphipod gut (2), as well as hadal sediments, including Mariana Trench, Steep wall site (Mariana Trench), Challenger Deep (Mariana Trench), and Basin site adjacent to Mariana Trench (3). A Kruskal-Wallis test was performed on the ARG abundances calcaulated from the metagenomes across these habitats.


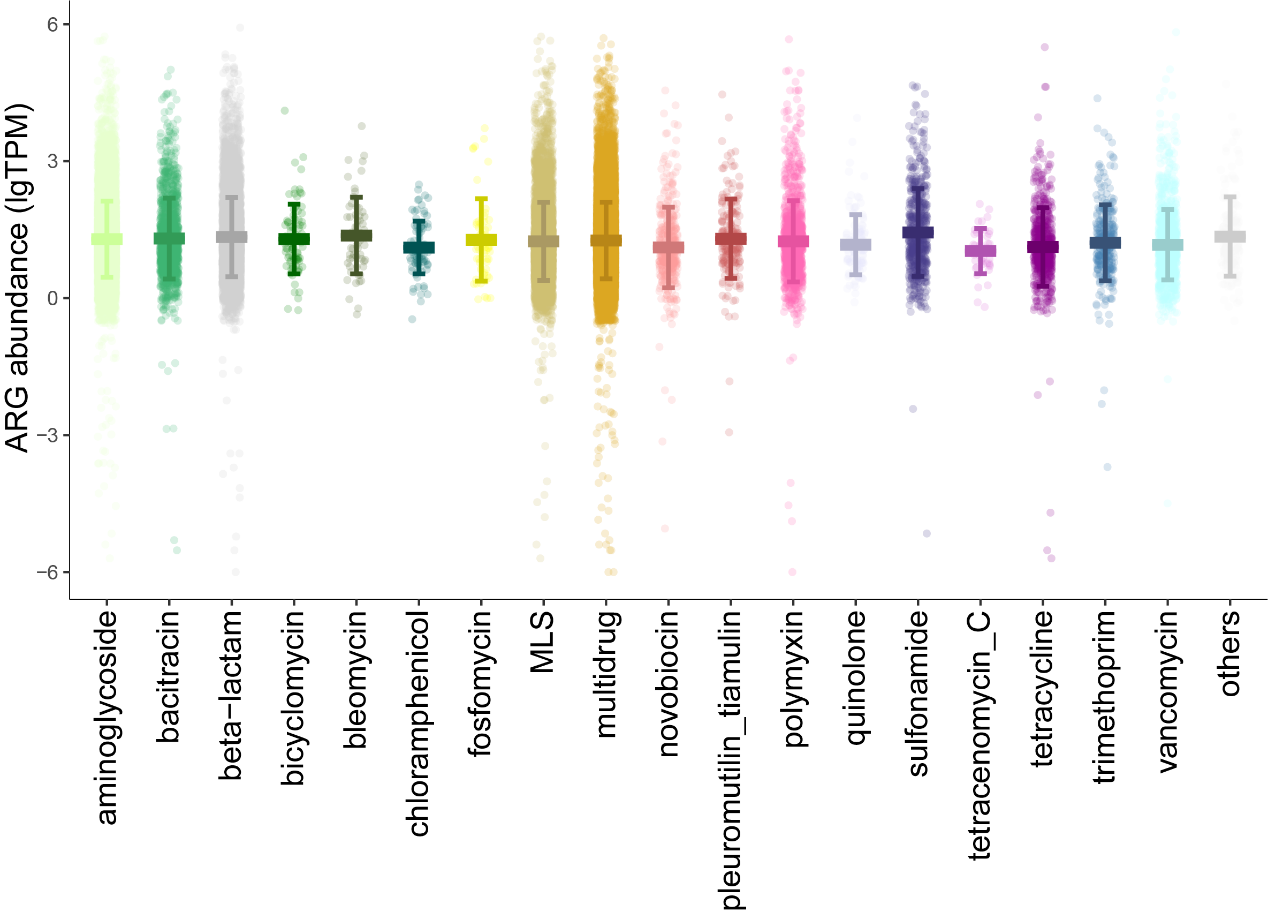


**Fig. S9. Transcript abundances of ARGs across different types from 33 cold seep sediment samples.** Each point represents the transcript abundance of a ARG at a cold seep site. Vertical bars indicate the minimum and maximum ARG transcript abundances. Transcript abundances are represented in units of transcripts per million (TPM), with values shown on the graph as log10(x+1). Details are shown in **Table S8**.


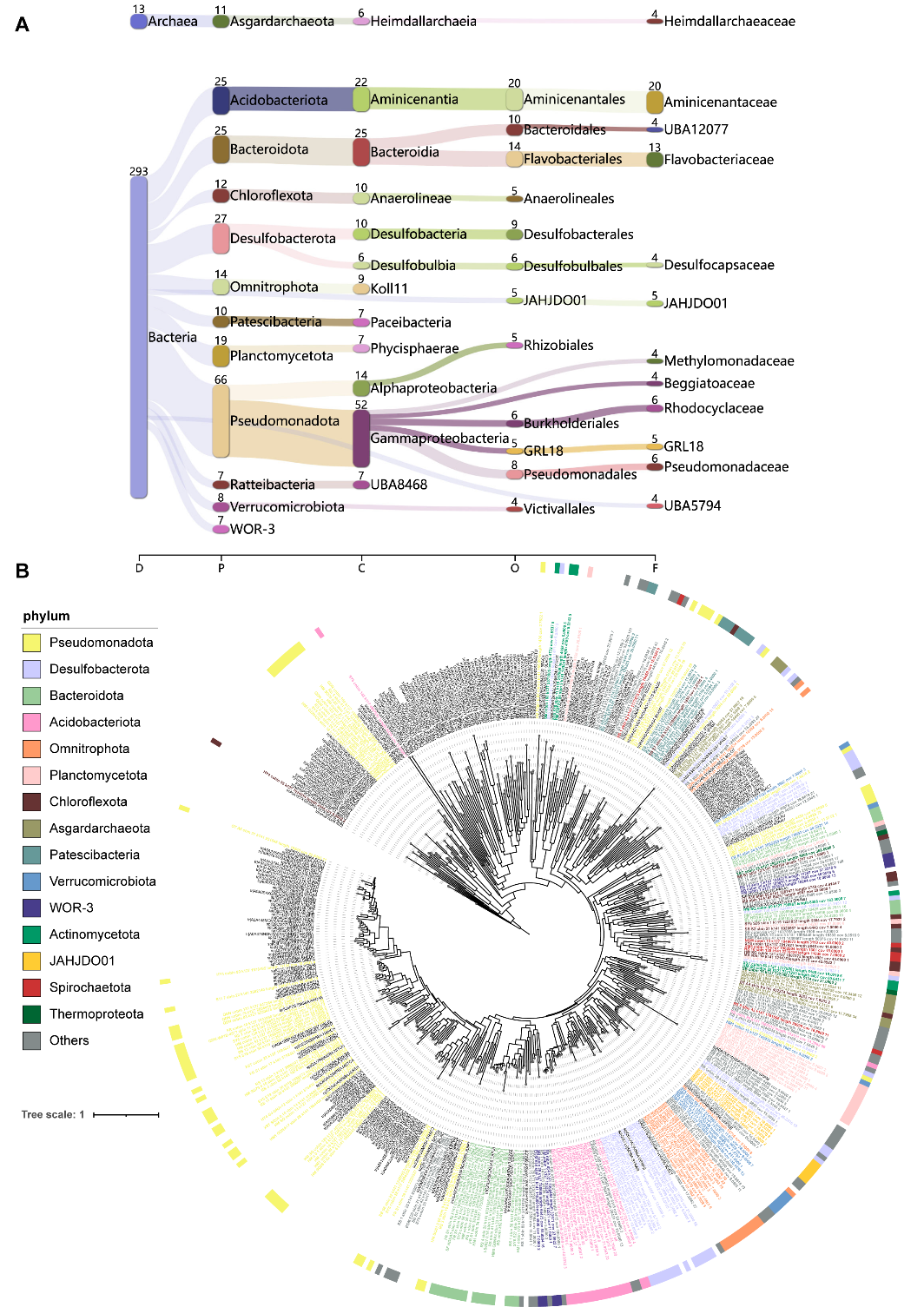


**Fig. S10. *msbA* identified from 3,164 cold seep MAGs. (A)** Sankey diagram illustrating the taxonomic distribution of MAGs containing the *msbA* gene. **(B)** A maximum-likelihood phylogenetic tree of *msbA* genes identified in cold seep MAGs (n = 260) and reference sequences (n = 216). Reference structures are colored in black.


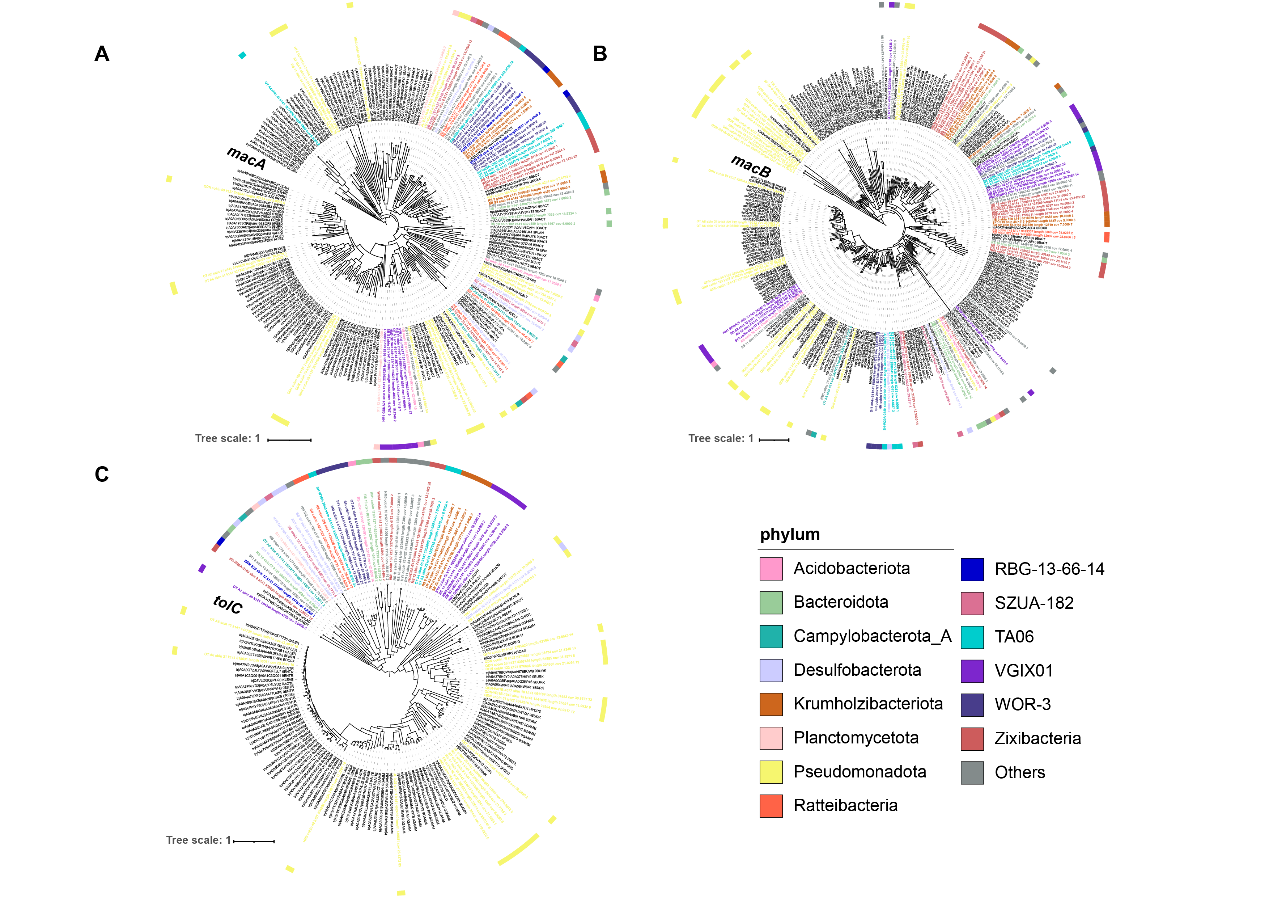


**Fig. S11. The phylogenetic trees of *macA*, *macB*, and *tolC* genes.** Maximum-likelihood phylogenetic trees are shown for **(A)** *macA*, **(B)** *macB*, and **(C)** *tolC* genes. The trees include genes identified in cold seep MAGs (n = 81 for *macA*, 109 for *macB*, 65 for *tolC*) and reference sequences (n = 152 for *macA*, 154 for *macB*, 97 for *tolC*). Reference structures are colored in black.


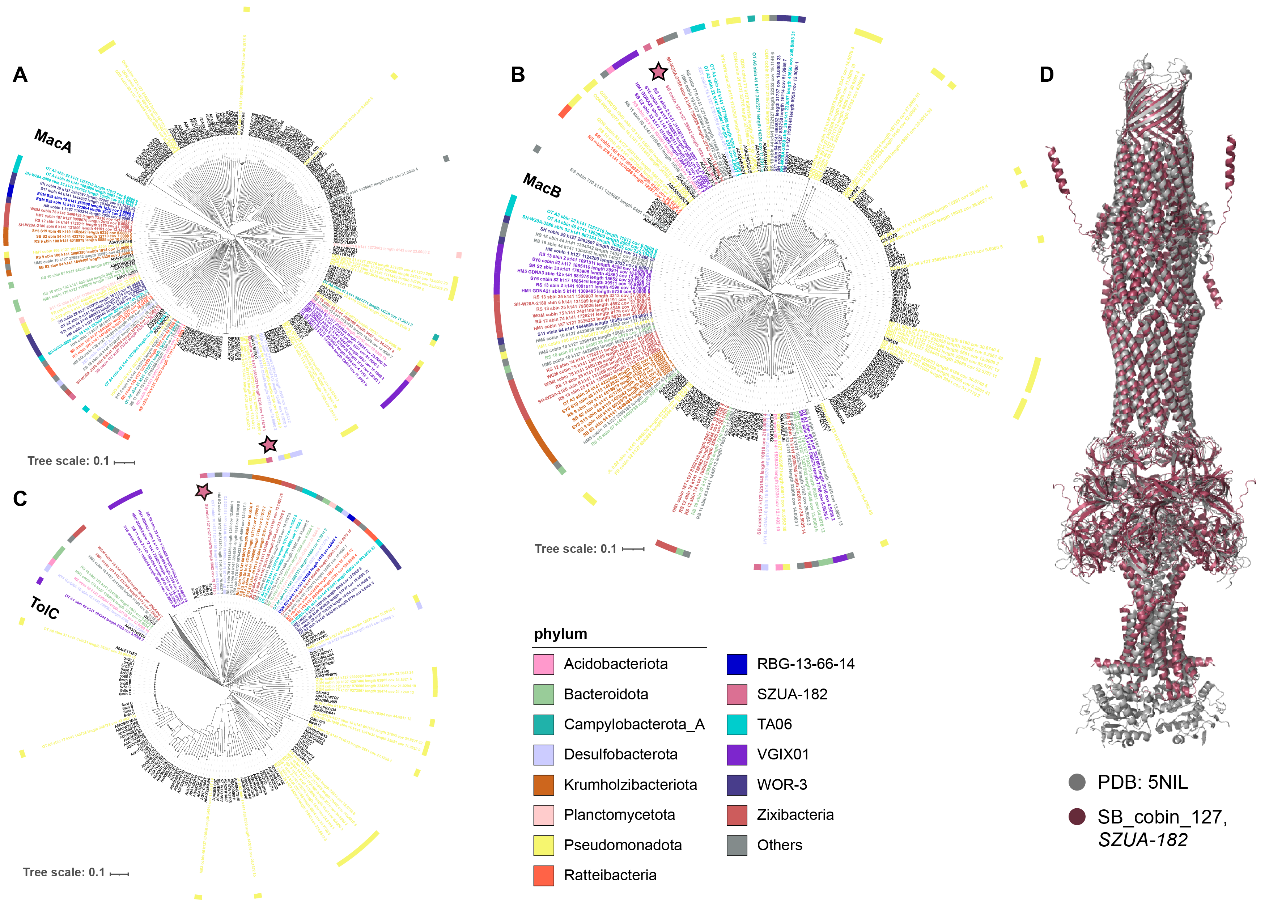


**Fig. S12. Trees and alignment of the MacAB-TolC pump. (A-C)** Structural trees of the MacAB-TolC pumps. The structures of *macA*, *macB* and *tolC* genes were predicted by ESMFold. The reference structures were downloaded from the AlphaFold Protein Structure Database (AlphaFoldDB) and the Protein Data Bank (PDB). Structural tree of MacA, MacB and TolC predicted by ESMFold (n = 81 for MacA, 109 for MacB, 65 for TolC) and the reference structures (n = 141 for MacA, 115 for MacB, 90 for TolC) downloaded from the AlphaFold Protein Structure Database (AlphaFoldDB) and the Protein Data Bank (PDB). **(D)** The predicted structure of this efflux pump (comprising 11 proteins) was aligned with the structure from *Escherichia coli K-12* (5NIL). Reference structures are colored in black.


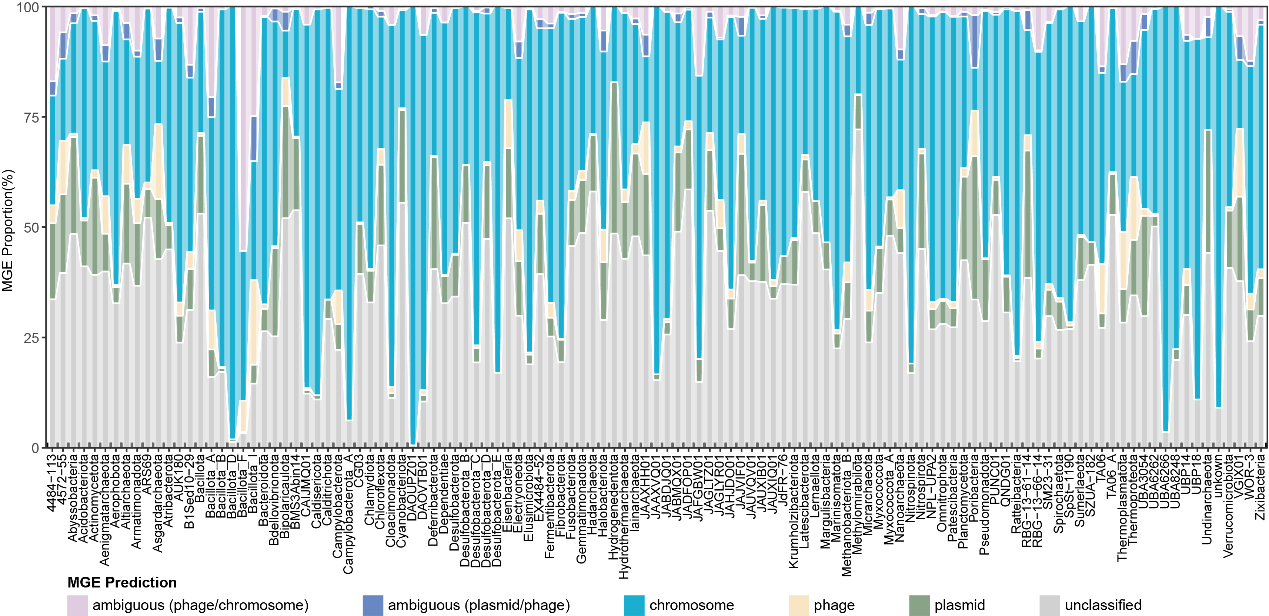


**Fig. S13. Relative proportion of different VF positions detected in 3,164 cold seep MAGs across different phyla.** The positions of VFs include ambiguous (phage/chromosome), ambiguous (plasmid/phage), chromosome, phage, plasmid, and unclassified.


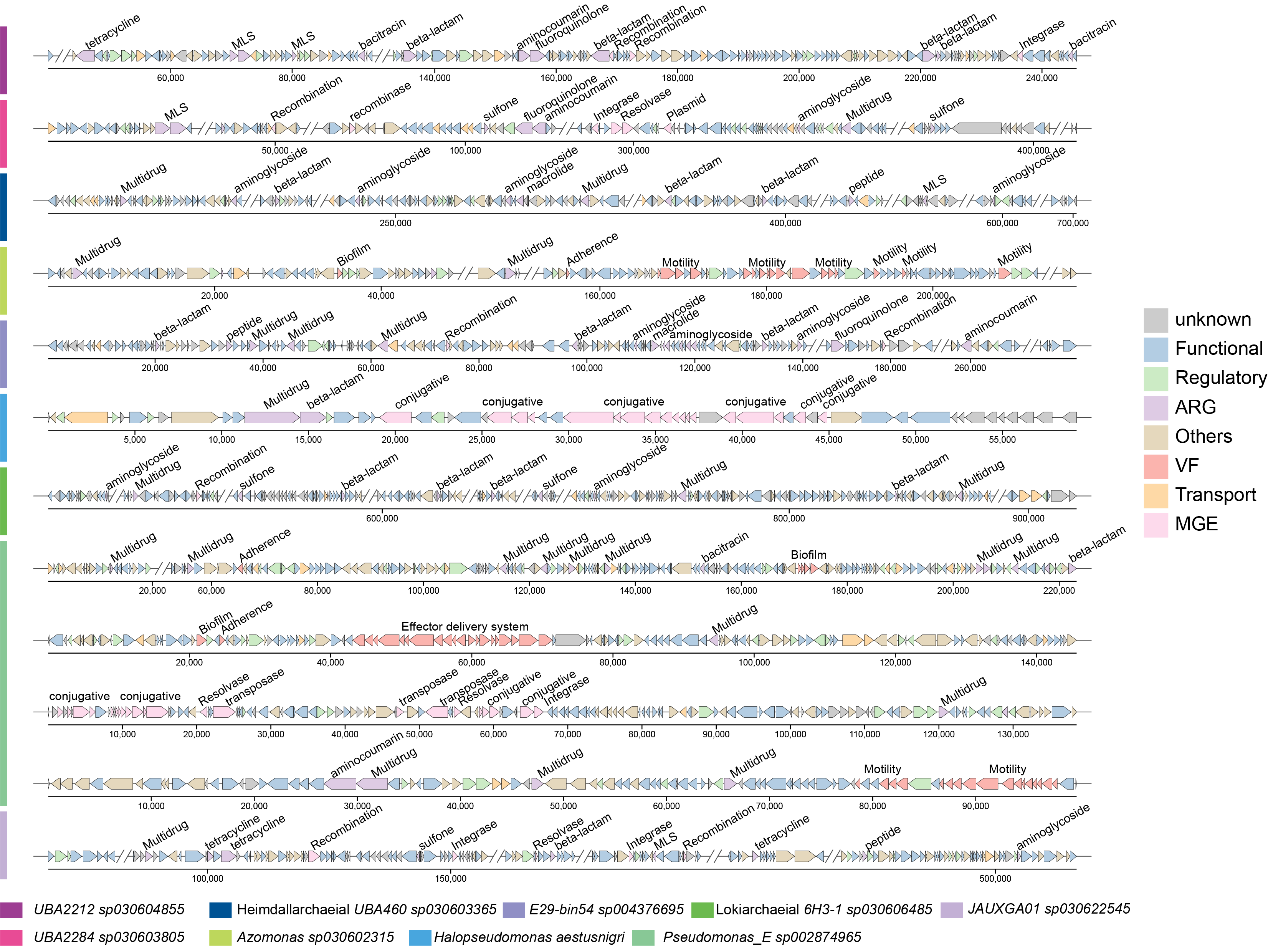


**Fig. S14. Co-localization of VF genes and ARGs with neighboring genes.** Functional annotations are color-coded, representing categories such as functional genes, transport, regulatory elements, VFs, ARGs, MGEs, and others. The colors on the left indicate the species to which each contig belongs.


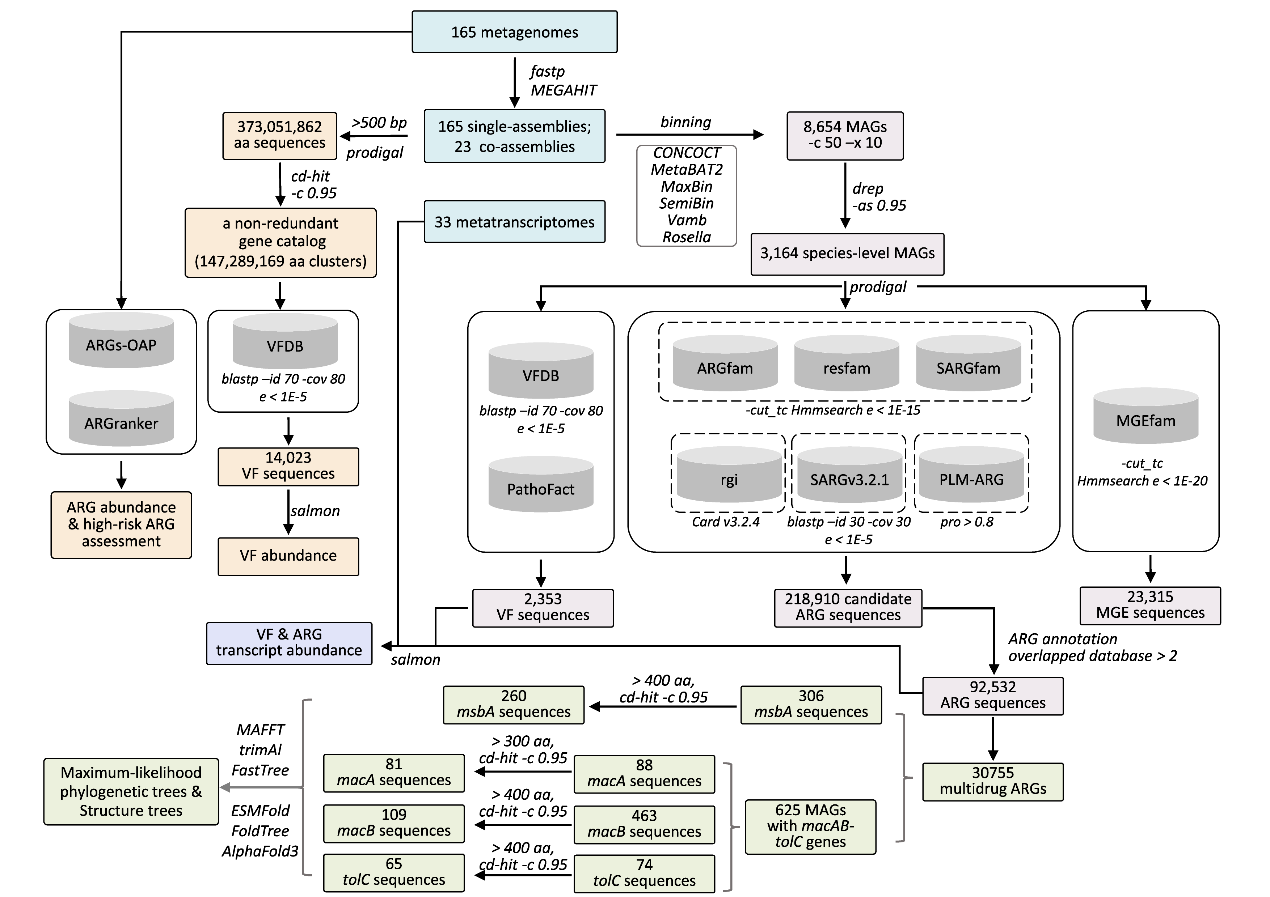


**Fig. S15. Bioinformatics workflow for analysis of virulome and resistome in cold seep sediments.** The workflow consists of: (1) metagenome processing to generate a non-redundant gene catalog and species-level non-redundant MAGs, (2) annotation of virulence factors (VF), antibiotic resistance genes (ARG), and mobile genetic elements (MGE), (3) calculation of VF and ARG transcript abundance, and (4) phylogenetic analysis of *msbA* and *macAB-tolC*. For detailed methods, refer to the Materials and methods section of the main text.

**References:**

1. Han Y, Zhang C, Zhao Z, Peng Y, Liao J, Jiang Q, Liu Q, Shao Z, Dong X. 2023. A comprehensive genomic catalog from global cold seeps. Sci Data 10:596.

2. Lee J, Ju F, Beck K, Bürgmann H. 2023. Differential effects of wastewater treatment plant effluents on the antibiotic resistomes of diverse river habitats. The ISME Journal 17:1993-2002.

3. He L, Huang X, Zhang G, Yuan L, Shen E, Zhang L, Zhang X-H, Zhang T, Tao L, Ju F. 2022. Distinctive signatures of pathogenic and antibiotic resistant potentials in the hadal microbiome. Environmental Microbiome 17:19.
